# Supplementary material for: Minimal specifications for non-human primate MRI: Challenges in standardizing and harmonizing data collection
Source: Neuroimage. Author manuscript; Available in PMC 2021 Nov 16. (PMC8594288; doi:10.1016/j.neuroimage.2021.118082)
Supplement: 1 [file NIHMS1749379-supplement-1.pdf]

Supplementary Information

Minimal Specifications for Non-Human Primate MRI: Challenges in Standardizing and Harmonizing Data Collection

Joonas A. Autio[1], Qi Zhu[2,3], Xiaolian Li[2], Matthew F. Glasser[4,5], Caspar M. Schwiedrzik[6,7], Damien A. Fair[8], Jan Zimmermann[8], Essa Yacoub[8], Ravi S. Menon[9], David C. Van Essen[5], Takuya Hayashi[1], Brian Russ[10,11,12,*], Wim Vanduffel[2,13,14,15,*]

[1]Laboratory for Brain Connectomics Imaging, RIKEN Center for Biosystems Dynamics Research, Kobe, Japan
[2]Laboratory for Neuro- and Psychophysiology, Department of Neurosciences, KU Leuven Medical School, Leuven 3000, Belgium
[3]Cognitive Neuroimaging Unit, INSERM, CEA, Université Paris-Saclay, NeuroSpin Center, 91191 Gif/Yvette, France
[4]Departments of Radiology and [5]Neuroscience, Washington University School of Medicine, St. Louis, MO, USA
[6]Neural Circuits and Cognition Lab, European Neuroscience Institute Göttingen – A Joint Initiative of the University Medical Center Göttingen and the Max Planck Society, Grisebachstraße 5, 37077 Göttingen, Germany
[7]Perception and Plasticity Group, German Primate Center – Leibniz Institute for Primate Research, Kellnerweg 4, 37077 Göttingen, Germany
[8]Center for Magnetic Resonance Research, Department of Radiology, University of Minnesota, Minneapolis, MN, USA
[9]Centre for Functional and Metabolic Mapping, Western University, London, ON, N6A 5B7
[10]Department of Psychiatry, New York University Langone, New York City, New York, USA
[11]Center for the Biomedical Imaging and Neuromodulation, Nathan Kline Institute, Orangeburg, New York, USA
[12]Department of Neuroscience, Icahn School of Medicine, Mount Sinai, New York City, New York, USA
[13]Leuven Brain Institute, KU Leuven, Leuven 3000, Belgium
[14]Athinoula A. Martinos Center for Biomedical Imaging, Massachusetts General Hospital, Charlestown, MA 02129, USA
[15]Department of Radiology, Harvard Medical School, Boston, MA 02144, USA

Incl. **Supplementary Figures S1-9**

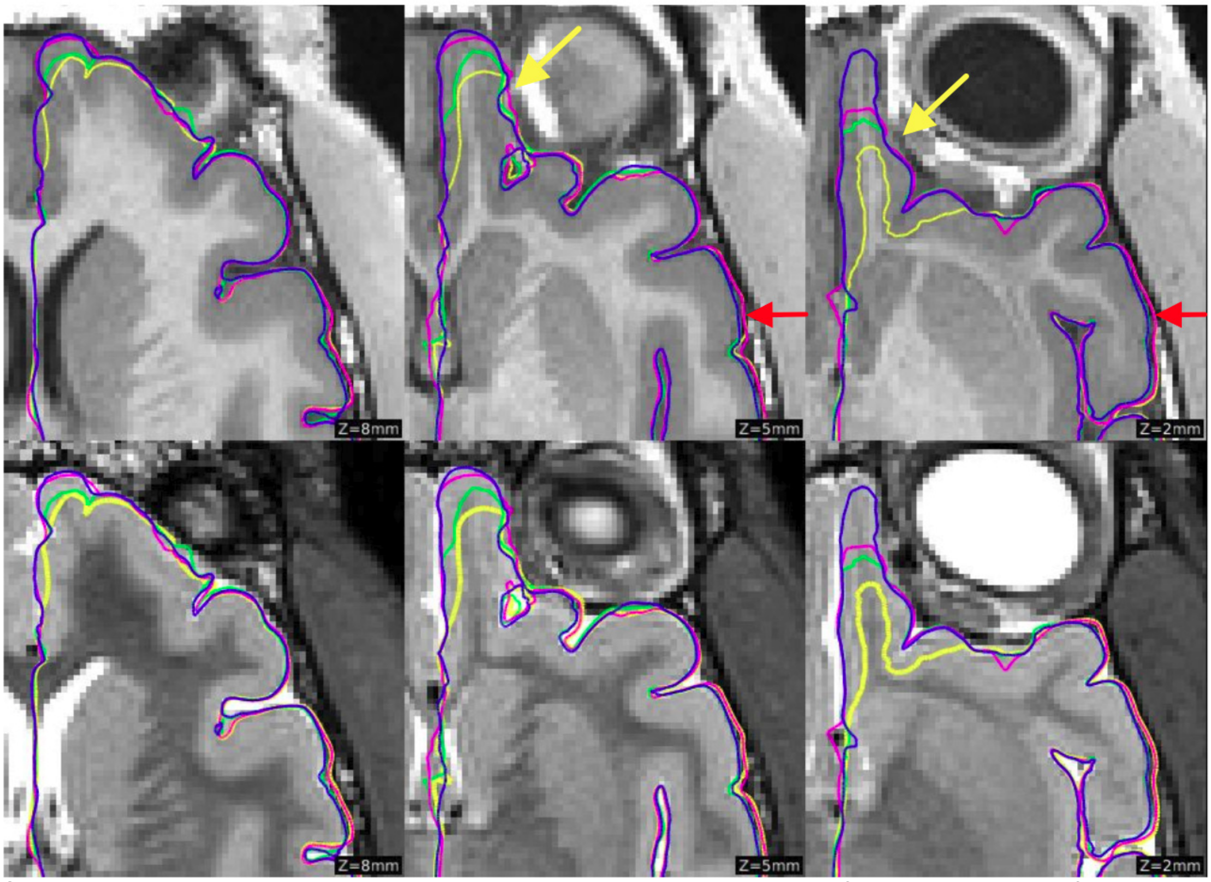

Supplementary Figure 1. Prescan normalization and T2w images improve cortical surface placement. Pial surface estimation using (i) non-prescan normalized T1w (yellow contour), (ii) prescan normalized T1w (fuchsia), (iii) non-prescan normalized T1w and T2w (lime) and (iv) prescan normalized T1w and T2w (blue) images. Upper row shows T1w and bottom row T2w images. Prescan normalized T1w and T2w images provided most robust pial surface estimation. Non-prescan normalized T1w and T2w images may result in mistakes between WM and GM segmentation and subsequent surface estimations (yellow arrow). Note that without the T2w image the pial surface estimation extends beyond grey matter to dura mater (red arrow). Data was analyzed using NHP-HCP pipeline (Glasser et al., 2013, Donahue et al., 2018, Autio et al., 2020).

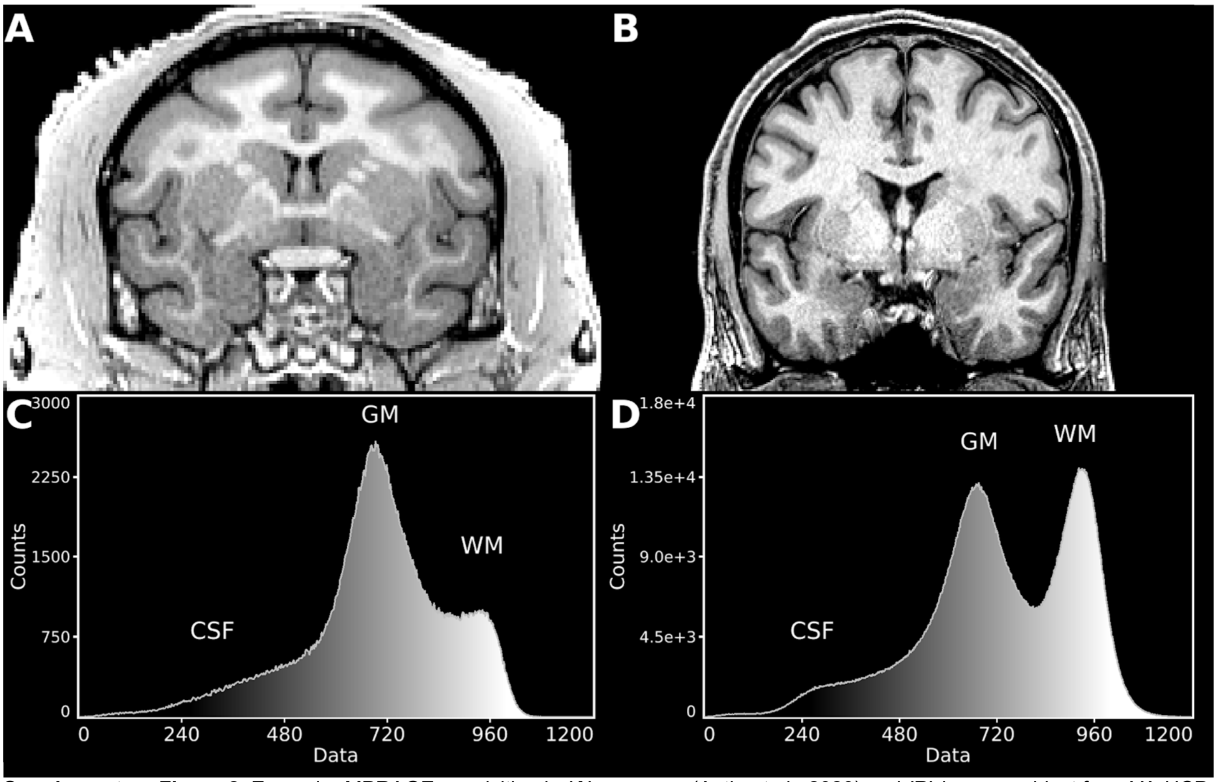

Supplementary Figure 2. Exemplar MPRAGE acquisition in **(A)** macaque (Autio et al., 2020) and **(B)** human subject from YA-HCP at 3T (Glasser et al., 2013). Image histogram in **(C)** macaque and **(D)** human. Note the distinct grey matter (GM), white matter (WM) and cerebrospinal fluid (CSF) peaks. In the human, inversion time (TI) was varied from 900ms to 1100ms in 100ms increments and 1000ms was found to approximately split the difference between the white matter and CSF peaks. This maximizes intracortical contrast for myelin and improves the performance of FreeSurfer tissue segmentation. In macaque, TI=900ms replicates this finding. The histograms are from brainmasked, B_1- intensity bias corrected images.

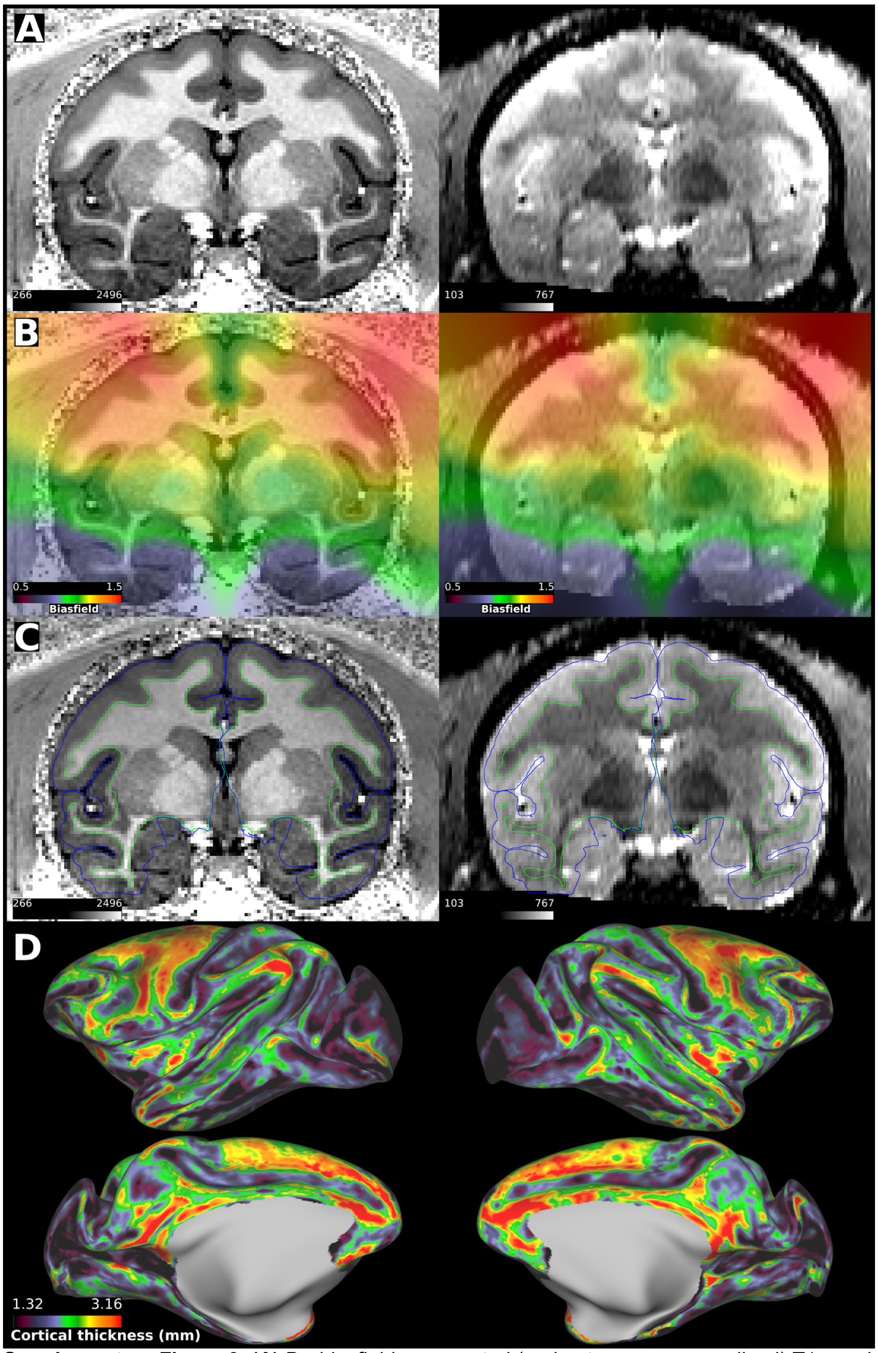

Supplementary Figure 3. (A) B_1- biasfield uncorrected (and not prescan normalized) T1w and T2w images. **(B)** Intensity biasfield, due to uncorrected B_1- and shared B_1+, estimated using a square root of product of T1w and T2w images. **(C)** Biasfield corrected T1w and T2w images. Note errors in pial surface estimations at more inferior parts of the brain and white surfaces proximal to insular cortices. **(D)** Cortical thickness displayed over inflated cortical midthickness surface. Data was acquired using an 8-channel surface RF transmitter and a 24-channel RF receive coil at 7T.

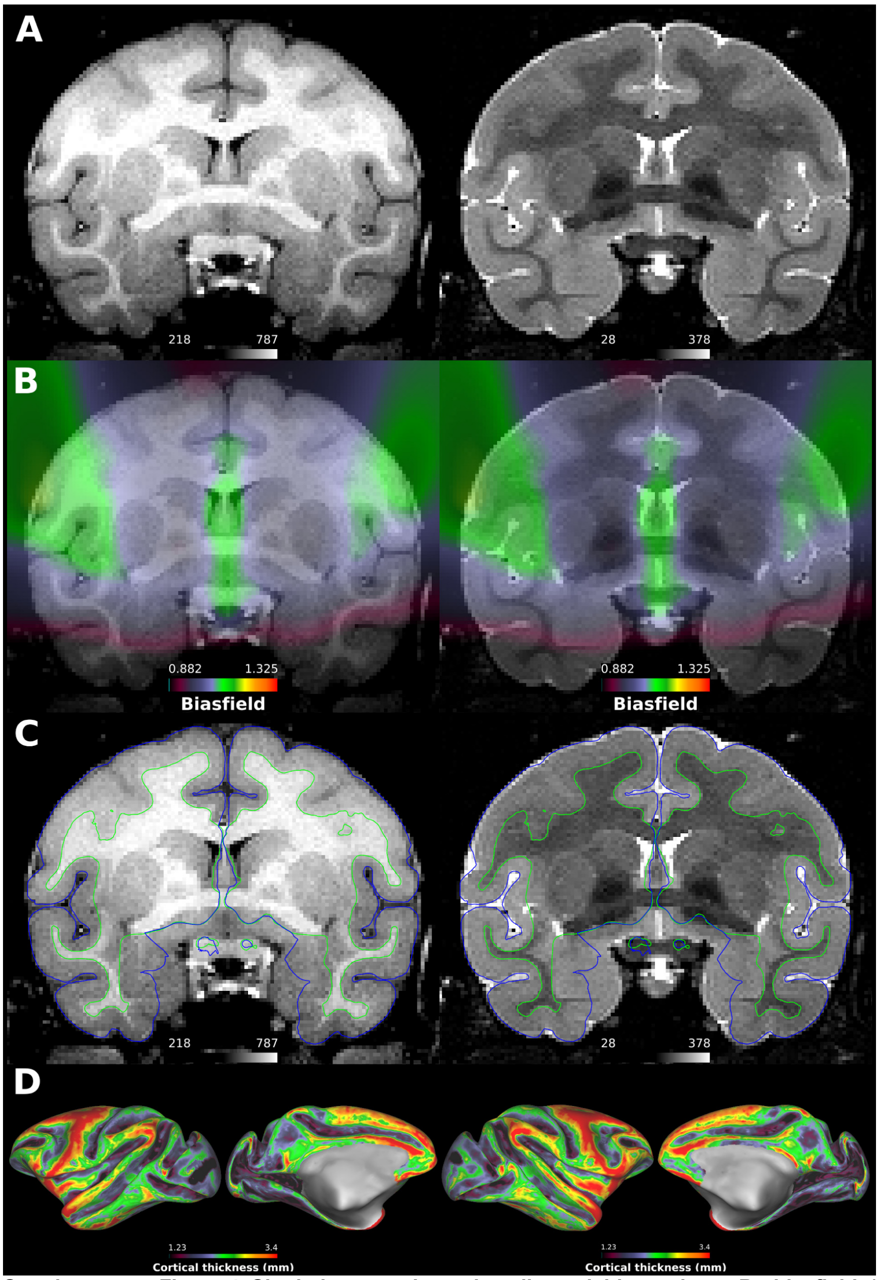

Supplementary Figure 4. Single-loop receive-only coil acquisition reduces B_1- biasfield. (A) B_1- biasfield uncorrected (and not prescan normalized) T1w and T2w images. **(B)** Intensity biasfield, due to uncorrected B_1- and shared B_1+, estimated using a square root of product of T1w and T2w images. Note that the B_1 biasfield is notably smaller in comparison with multichannel coil acquisitions (colormap is matched with Fig. 1b). **(C)** Biasfield corrected T1w and T2w images. **(D)** Cortical thickness displayed over inflated cortical midthickness surface. Data was acquired using a single-loop RF receive coil at 3T Siemens PRISMA at 0.4 mm isotropic resolution (Li et al., 2019; Zhu and Vanduffel, 2019). The data acquisition time for T1w and T2w images were 152 min and 123 min, respectively.

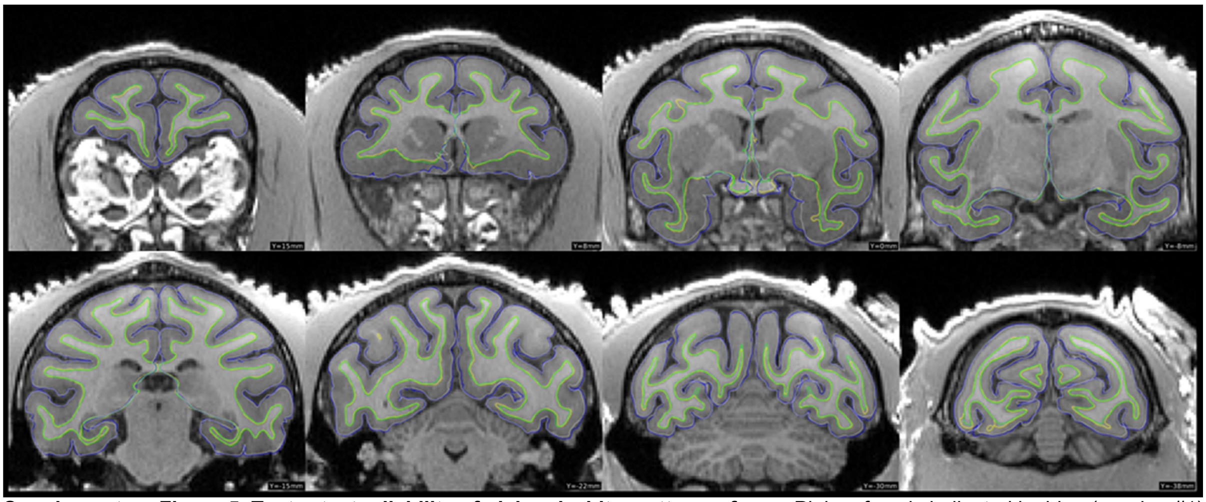

Supplementary Figure 5. Test-retest reliability of pial and white matter surfaces. Pial surface is indicated by blue (session #1) and silver (session #2) contours. White matter surface is indicated by yellow (session #1) and green (session#2) contours. Session #2 was acquired approximately one and a half months after session#1. Data were acquired using the Human Connectome Project (HCP)-style imaging and automatically processed using non-human primate HCP pipeline (Autio et al., 2020; Donahue et al., 2018; Glasser et al., 2013). Note the good reproducibility of cortical surfaces. Data available at https://balsa.wustl.edu/study/show/kNj6K

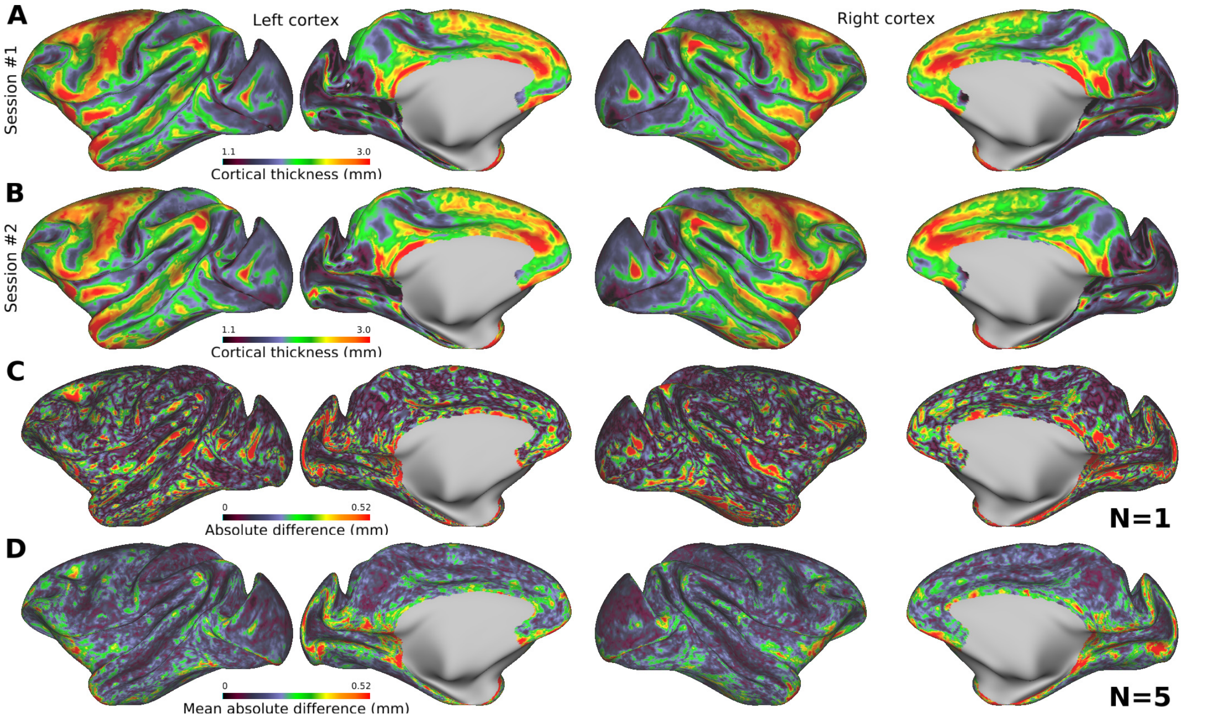

Supplementary Figure 6. Test-retest reliability of cortical thickness. (A) Session #1 and **(B)** session #2, acquired two weeks after session #1. **(C)** Absolute cortical thickness difference between the two imaging sessions. **(D)** Average absolute difference (N=5). Range scaled to 25% of median cortical thickness (~2.1 mm). Dense cortical thickness measures were reproducible with Pearson correlation coefficient of 0.90 ± 0.02. However, substantial errors, up to 0.6 mm, are identified in some of the geometrically more challenging regions including parahippocampal complex, ventral insula, and medial visual areas.

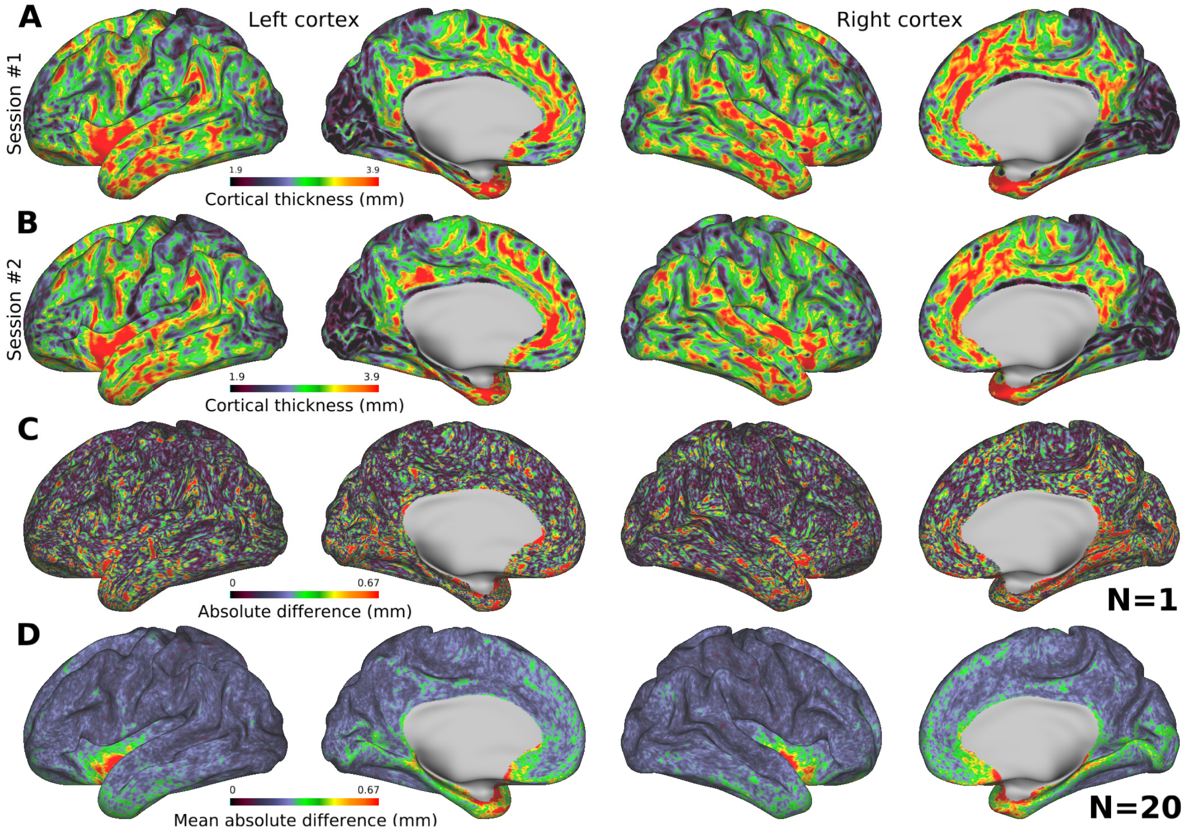

Supplementary Figure 7. Test-retest reliability of cortical thickness in YA-HCP. (A) Session #1 and **(B)** session #2. **(C)** Absolute difference in cortical thickness between the two imaging sessions. **(D)** Average absolute difference (N=20). Range scaled to 25% of median cortical thickness (~2.7 mm). Dense cortical thickness reproducibility was good with Pearson correlation coefficient of 0.82 ± 0.03. Errors are identified in some of the geometrically more challenging regions similar to macaque monkeys (see Supp. Fig. 6d).

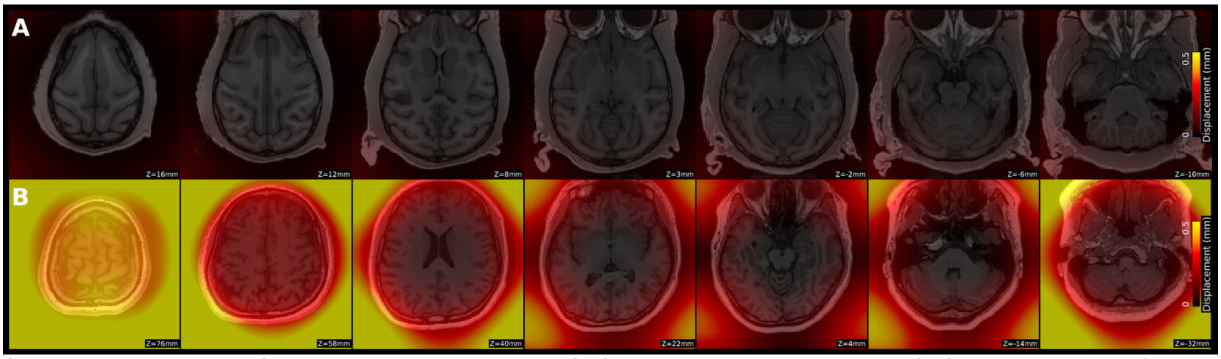

Supplementary Figure 8. Gradient distortion correction (GDC). (A) Macaque and **(B)** human GDC corrected images. Colorbar indicates the (absolute, Euclidian) displacement (units in mm) due to gradient nonlinearities. Note that the displacement in macaque (maximum < 0.03 mm) is an order of magnitude smaller in comparison to human (up to 0.4 mm). The gradient nonlinearity field is from a Siemens 3T PRISMA equipped with XR 80/200 gradient system.

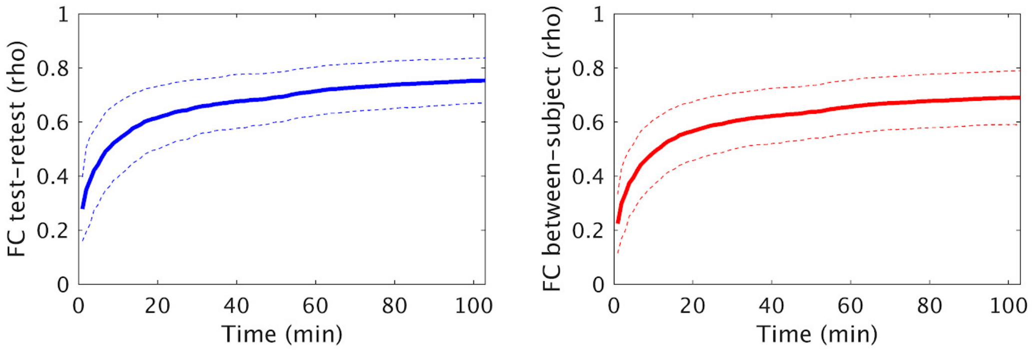

Supplementary Figure 9. (A) Within-subject test-retest (blue line) and **(B)** between-subjects (red line) resting-state functional connectivity (FC) correlation matrix (Pearson's r) estimates converge towards longer scan durations. The solid lines indicate average Spearman's Rank correlation and dotted lines indicate the standard deviation (N=5). The RIKEN macaque data was FIX-cleaned and parcellated using M132 atlas (Markov et al., 2014).
